# The Chicago Gynæcological Society, Meeting January 15th

**Published:** 1886-03

**Authors:** 

**Affiliations:** 2330 Indiana Avenue


TRANSACTIONS OF THE CHICAGO GYNÆCOLOGICAL SOCIETY.

*LXII Meeting, Friday, January 15th, 1886.*

I.—EARLE. *The Watery Discharges of Pregnant Women.*

II.—DOERING. *Report of a Case of Hydatiform Pregnancy.*

III.—WAXHAM. *Intubation of the Larynx, with History of Cases.* (*Inaugural Thesis.*)

The PRESIDENT, PROFESSOR DANIEL T. NELSON, M. D., in the chair.

I.—PROFESSOR CHARLES WARRINGTON EARLE read a paper entitled,

"THE WATERY DISCHARGES OF PREGNANT WOMEN."

Mrs. F. K. consulted me for a profuse watery discharge which had taken place several times during her pregnancy, commencing at the third month. She was the mother of three children, and had always been free from any marked pelvic disease. The first discharge was clear and watery, and she estimates the quantity at about two quarts. This came away in a gush, most of it being discharged at once, although there was a slight loss for some days thereafter. At first it was thin and clear, then slightly thicker, of the color of weak coffee. These discharges seemed to occur every two or three weeks, and were frequently attended with considerable pain. There was a decided diminution in the size of her abdomen after each discharge.

On October 30th, I found her in great pain, and an examination demonstrated that the fœtus was very low in the pelvis and apparently not surrounded with any *liquor amnii*. The *os uteri* was neither soft nor dilated. She was ordered anodynes and to remain in bed. On the 7th of November, I again saw her, and found she had been having more or less pains since my previous visit. There was no dilation. Two days after, however, she was delivered, her gestation having lasted about 200 days. The child lived about one hour. She made a good recovery and resumed her place in her family in the course of two weeks.

Mrs. M., 27 years old; in her ninth pregnancy. At the end of five months she commenced to have a flow of fluid which continued until the end of the seventh month, when she gave birth to twins, one living and the other dead. There was no escape of *liquor amnii* at her confinement. The same lady in her eleventh pregnancy commenced to lose fluid at the end of the seventh month, which continued until the completion of the full term, when she gave birth to a healthy child. She had what her attendants called a dry labour.

Mrs. D. W. R., aged 31, the mother of nine children, has been pregnant since the 1st of July, 1885. On November 20th, she said to a friend who was at her bedside that she was flowing and asked to be supplied with a napkin. A sheet folded and placed under the patient was thoroughly saturated with fluid; the discharge being equal probably to at least two pints. She had severe pains, which simulated those of labour, lasting a few hours. On December 15th, she had a similar discharge. The future of this case is yet to be decided.

*Frequency.*—These cases evidently take place with more frequency than we have, up to this time, supposed; but the older obstetric authors have noticed peculiarities of this kind, and given very fair descriptions of the complication.

SMELLIE says (page 179, Vol. II.): "Dribbling of fluid may go on for weeks, but a sudden gush is invariably followed by parturition; the longest interval between a sudden gush and labour being seven days." In this he is certainly mistaken, as the history of many recorded cases and some of mine will demonstrate.

DENMAN, 1815, says: "Instances have been recorded in which the waters of the ovum are said to have been voided as early as the sixth month of pregnancy without prejudice either to the child or the mother. The truth of these reports seems to be doubtful, because where the membranes are intentionally broken, the action of the uterus never fails to come on. A few cases of this kind, somewhat similar, have occurred to me. A discharge of colorless fluid takes place, daily, from the vagina for several months preceding labour, which is due to the rupture of some lymphatic. Such labours are usually premature and the fœtus small."

The same authority also cites a case where, after the delivery of the placenta, several pints of lymph were discharged.

BURNS, 1822, page 238, says that the discharges of watery fluid from the vagina are not infrequent, and generally depend upon the secretion of glands about the cervix; the rupture of lymphatics, or from fluid collected between the chorion and amnion, or water from blighted ovum in the case of twins.

DR. PENTLAND relates a case where coughing produced a discharge, the water being discharged at the fourth month; but labour only occured at full term.

MERRIMAN, in his work entitled "Difficult Parturition," 1826, relates the case of a lady—six months pregnant—from whom a profuse, watery discharge occurred. She summoned a physician, who assured her that if pains came on she would soon be delivered, She continued, however, to the end of pregnancy, having a profuse discharge each day. At full term she was delivered, her attending physician rupturing a bag of waters, which appeared in no way different from usual cases. No opening was discoverable in either the placenta or the membranes, and he concluded that the discharge must have come from the outside of the membranes.

CHAILLY, edited by BEDFORD, 1844, gives a rather full account of hydrorrhœa, the description not being different from those I have already related. He says, however, that these discharges are more frequent than are generally supposed, but makes the erroneous statement that in nearly all these cases pregnancy is carried along to its full term.

Nearly all modern authors devote a short section to the consideration of this subject, giving different names, as their ideas of its origin and pathology are different.

Three separate pathological conditions seem to be, in many cases, confounded, and I see no way by which a differentiation can be made.

1st. A discharge of the *liquor amnii.*

2d. Discharges from increased glandular action.

3d. A possible collection of fluid between or outside of the membranes, and its irregular evacuation.

In my teachings I have been in the habit of speaking of hydrorrhœa, but never, up to a few months ago, had I seen a marked case. A study of this case with others collected from my own experience, and the perusal of the article written by DR. THOMAS C. SMITH, of Washington, D. C., which appeared in the *American Journal of Obstetrics* in May, has caused me to go over the subject carefully and to present what I can obtain from the authorities in regard to these peculiar discharges.

Great numbers of cases have been recorded, but no one, up to this time, has demonstrated conclusively the source of the flow.

*The etiology* of these discharges has been the subject of very different opinions by different obstetric authors. CHAILLY says that authors have attempted to show that these discharges are due to the accumulation of fluid between chorion and amnion; to rupture of lymphatic vessels; to transudation through amniotic membranes; to rupture of the membranes at some remote point from the orifice of the uterus, and finally, to dropsy of the womb.

LUSK says the pathological processes involved in the disease are vascularity, hyperæmia and hypertrophy of the interstitial connective-tissue, and of the glandular elements of the decidua.

BARNES, in the "System of Obstetric Medicine and Surgery," 1885, says in regard to these discharges, without entering into a critical discussion of the several theories, that it seems to be well established that there are five sources rom which this fluid may come:

1st. A discharge from the cervical canal.

2d. The decidual origin.

3d. Transudation through the amniotic membranes.

4th. Hydatidiform degeneration of the ovum.

5th. Cauliflower excrescences.

The *Differential Diagnosis* must rest between the following similar discharges:

I. From the discharge from hypertrophied cervical glands.

II. Fluid collecting between chorion and amnion, occurring only once.

III. Escape of fluid from amniotic cavity.

I. The fluid escaping from the hypertrophied glands must be small in quantity, and we would expect that it would continue for a considerable length of time. There would be no diminution in the amount of *liquor amnii* and the child would be found floating in the usual amount of fluid.

II. If the fluid collected between any of the membranes, and adhesive inflammation surrounding it followed, a considerable amount of fluid might collect, and the discharges would be considerable at once, and might or might not be repeated. In such a case there would be no evidence of escape of true amniotic fluid, although there might be a lessened size of the abdomen.

III. Where the *liquor amnii* escapes there would be a greater tendency to uterine contractions; a more perceptible diminution in the size of uterine tumor, and a microscopical or chemical examination would certainly reveal some evidence of urine, as we know this exists in variable quantities in the *liquor amnii.*

Transudation through the amniotic membrane, although recently noticed by BARNES, and mentioned by older authors, would give rise to the discharge of a very small amount of fluid.

This could hardly be differentiated from a slight discharge taking place from the cervical glands. Fluids discharged from hydatidiform degeneration of the chorion or from cauliflower excrescence, would be so associated with the diseases which cause them that the diagnosis would not be difficult.

*Prognosis.*—As far as my observation goes, the life of the woman is not jeopardized, but she suffers from the constant discharge and becomes anæmic. The pain is sometimes severe, as I have before remarked, and the patient is full of gloomy forebodings and anxious in regard to the final result.

The fœtus is usually born prematurely, and, in many cases, only lives a short time.

The treatment must necessarily be very simple—rest and anodynes being about all that can be suggested.

DISCUSSION.

DR. H. P. MERRIMAN: MR. PRESIDENT, I had one case of this kind about a year ago. The woman had a sudden gush of water when she was not quite five months pregnant. I thought it might presage labour, and told her to let me know of any symptoms of labour,—that I expected it would come on. But she felt better after having had the gush of water. She had, in the course of two or three weeks, another, and said she could tell when they were coming on, because she felt so full before they came. When the second came I began to think that perhaps she was not going to have labour at the present time after all; that it probably was not a loss of the amniotic fluid, and I examined her and found the os not dilated. I could feel, however, by carefully introducing my finger, that there was water still remaining there,—the amniotic bag remaining apparently intact. I gave her opiates, thinking that labour might possibly be prevented. She went along for nearly a month after that, before she finally miscarried. She had three separate gushes of water at intervals of two or three weeks before her miscarriage finally came on. The fœtus had perhaps a little over six months of intra-uterine life at the time of its expulsion.

It strikes me that we might learn by careful examination of the placenta and membranes after delivery, a great deal more than we have yet learned about this subject. I cannot help thinking that there must be some defect in the fœtal envelopes to have a thing like this occur. It could not have been a rupture of the amnion, but there may have been a separation between the amnion and the chorion, as I have seen in one other case in my own practice, in which the infant or fœtus enveloped in the amnion came away, leaving the chorion within the uterine cavity. And we had a similar case presented to the Society a year ago, by Doctor SAWYER. The amnion had been separated from the chorion, and came away intact by an effusion of liquid between the chorion and amnion. Now, if that takes place, why of course there may be a separation in part and then adhesion again after the occurrence of the rupture. Any gush of this kind indicates, to me at least, some disturbance of the fœtal envelopes, either of the chorion or amnion, or a cystic degeneration of the placenta; and it strikes me that in every case of this kind the placenta and membranes ought to be carefully observed after the delivery, to see what pathological cause brought on the abortion.

I would like to state, in addition to my case, that the woman finally had her miscarriage quite suddenly. I was not present, and another physician was called.

THE CHAIRMAN: I would like to ask a question as to whether there is any specific cause operative in the production of these cases? Whether syphilitic or gonorrhœal infection may have anything to do with it, and also whether inflammation of the mucous membrane of the uterus precedes these causes? Is it, in other words, an acute or chronic inflammation of the mucous membrane that causes it?

DR. HENRY T. BYFORD: I have nothing to add, except that Dr. C. R. PARKE, of Illinois, reported a case to me, in which the discharge of the *liquor amnii* took place, labour pains came on, and the umbilical cord became prolapsed. He replaced the cord and gave ergot. As labour did not progress, he finally gave morphia and quieted the pains. In three months the woman was delivered of a living child; mother and child did well.

DR. H. P. NEWMAN: I saw a single case; the discharge however, was greater than in the cases related, and came on about six weeks previous to the abortion; the membranes were not examined.

PROFESSOR W. W. JAGGARD said that he had listened to the reading of Dr. EARLE's paper and the discussion with great interest. He could not, however, agree with the author of the paper in considering the pathology of *hydrorrhœa uteri gravidi* as obscure and confused in all its details. CARL BRAUN (*Zeitschr. d. Ges. d. Wiener Aerzte, 1858, No. 17, p. 257*) and C. HENNIG (*Der Katarrh der inneren weiblichen Geschlechtstheile, Leipz., 1862, p. 48*) had clearly and distinctly described the pathological anatomy of the condition. Chronic decidual endometritis may terminate in the formation of new connective tissue, or may manifest itself by the production of a yellow, sero-albuminous fluid, variable in quantity, which accumulates between *decidua vera* and *reflexa*, or when *vera* and *reflexa* are united, between decidua and chorion. CARL BRAUN accordingly considers the condition to be a *serous endometritis*. HENNIG aptly terms it *catarrhal decidual endometritis*. Catarrhal decidual endometritis must be distinguished from collections of fluid between the amnion and chorion, the so-called amnio-chorial water. BISCHOFF has designated the unorganized, albuminous fluid uniting chorion and amnion as the *tunica media*. The quantity of this fluid may increase abnormally, at the same time that its consistency is diminished. MCCLINTOCK describes a case, referred to by SPIEGELBERG, in which the amount of "amnio-chorial water" was so great as to simulate hydramnios. The "amnio-chorial water" may be discharged without the interruption of pregnancy, but then the discharge of fluid is not repeated as in the intermittent discharges of *hydrorrhœa uteri gravidi.* Labour always follows the rupture of the amniotic sac,—a fact which establishes the possibility of a differential diagnosis in the large majority of cases. It is unusual for labour to be prematurely induced by the discharge of the "amnio-chorial water," or collections of catarrhal secretions between chorion and decidua.

A condition strictly analogous to *hydrorrhœa uteri gravidi* is frequently observed in uterine fibroids. The intermittent discharge of a yellowish sero-albuminous fluid from the uterine cavity is a symptom of such frequent occurrence in this condition that attention is directed to it by most systematic writers.

With reference to the ætiology of *hydrorrhœa uteri gravidi*, there were several facts of practical import. Any antecedent endometritis,—gonorrhœal, syphilitic or of other origin,—is an adequate ætiological factor. Hydræmia appears to favor the development of the condition. The coincidence of hydræmia with catarrhal decidual endometritis would certainly indicate the exhibition of chalybeate tonics in the treatment of the latter affection.

He fully agreed with Dr. MERRIMAN in attaching great importance to the critical examination of the fœtal envelopes in order to clear up a doubtful diagnosis.

DR. EDWARD WARREN SAWYER called attention to the fact that watery discharges from the uterine cavity frequently occurred during the *puerperium.*

He thought that the condition, technically termed *hydrorrhœa gravidarum*, was due in all cases to the transudation of the amniotic fluid. This was the opinion ably advocated by CHARPENTIER.

PROFESSOR W. W. JAGGARD thought Dr. SAWYER had not quoted CHARPENTIER correctly. CHARPENTIER mentions STAPFER's recent monograph (*Thèse de concours*, 1880), in flattering terms; enumerates the various hypotheses proposed by a large number of observers, and says the German theory, already referred to, is the most probable.

PROFESSOR CHARLES WARRINGTON EARLE :

I have but very little to say, Mr. Chairman, in closing the discussion. It seems to me, however, that there is one thing, at least, that we should learn from our consideration of this subject this evening. It seems to be impossible for any one to determine the exact source from which a considerable amount of fluid is occasionally discharged from the vagina of a pregnant woman. We do not know whether this fluid comes from the amniotic cavity or external to it ; therefore, we should not give ergot or commence the dilatation of the *os uteri* after a watery discharge, believing that labour must come on, because from the testimony we have received here to-night, and from other evidence, it does seem that even if the *liquor amnii* is prematurely evacuated in a few cases, pregnancy may go on to full term.

My attention has been called to the phenomenon mentioned by Dr. SAWYER, and if I had not desired to make my paper as brief as possible, I should have spoken of the watery discharges which occasionally take place after labour. I have never seen a case, but it is mentioned in the literature, and it is believed by those who have written upon the subject that the fluid in these cases comes from either the large lymphatic vessels, or perhaps from a continuation of the same disease which produced the discharge before.

The Doctor is certainly not quite in accord with the majority of authorities when he says that the discharges of pregnancy always come from the cavity of the amnion.

DR. EDWARD WARREN SAWYER:

No; but the term "hydrorrhœa" should be reserved for that class of cases.

PROFESSOR EARLE:

This is not hydrorrhœa, as I understand it. This term should be applied to a discharge of fluid from outside of the amniotic membrane; perhaps not from outside of the chorion, but certainly from outside of the amnion.

II.—DR. E. J. DOERING read a paper entitled:

REPORT OF A CASE OF HYDATIFORM PREGNANCY.

After a brief discussion of the ætiology and pathology of cystic degeneration of the chorionic villi, Doctor DOERING related the history of the following case:

Mrs. W. D. P., a cultured lady, of slender physique, twenty-one years of age, was attended by me in labour fifteen months ago, and delivered by instruments of a healthy boy weighing ten pounds. Her general health has been good. She has had no miscarriages either previous to or since the birth of her child. Her last period occurred during the latter part of October, 1885. During the month of November the catamenia remained absent, which she attributed to a cold, the idea of pregnancy not occurring to her, as she had none of the usual symptoms. During the month of December, and particularly during the week preceding the holidays, she was on her feet constantly, although not feeling well, having sensations of chilliness, followed by a feeling of heat and general depression. On the Sunday before Christmas a slight and painless flow of blood commenced, believed by her to be the period, now four weeks overdue. The flow continued several hours and then ceased. On Christmas day, while seated at the dinner-table, she was suddenly attacked with a profuse hæmorrhage, the blood saturating the floor, and continuing until a degree of faintness was produced, in which condition I found her on my arrival a few minutes afterwards. The hæmorrhage, which had been entirely without pain, ceased suddenly. A careful examination confirmed my suspicion of pregnancy, although I was much surprised at the size of the uterus, corresponding to a four and one-half months' pregnancy, the fundus rising nearly midway between the symphysis pubis and the umbilicus. There being no further hæmorrhage, no pain and no dilatation of the os, an expectant plan of treatment was pursued by instructing the patient to keep in bed, enjoining absolute rest, and giving her a few doses of morphia. On the following night another hæmorrhage occurred, but of not much consequence, and requiring no interference. Two days later, on the morning of the 28th of December, another hæmorrhage took place, more copious than the last one, but still unaccompanied with pain. An examination showed slight dilatation of the os, but not sufficient to permit the recognition of the contents of the uterus. As the patient was beginning to show decided symptoms of anæmia, the vagina was tamponed and ergot administered to check the hæmorrhage and favor uterine contractions.

Uterine pains soon commenced, accompanied by considerable hæmorrhage; the os dilated fully one inch, the presenting part giving the sensation to the finger of a blood-clot. This was soon expelled in detached portions, and on removal from the vagina was readily recognized as a hydatiform mole, having all the characteristic appearance of a grape bunch, composed of a mass of translucent vesicles, about the size of currants, containing a clear, limpid fluid. After inserting two fingers into the uterus and emptying it as thoroughly as possible of all the diseased tissue, the hæmorrhage promptly stopped. The entire mass removed equaled about the size of a large orange. Some febrile reaction occurred, but for several days the temperature did not exceed 100½F°. and the pulse 95, the treatment consisting of quinine and ergot, internally, and the use of uterine and vaginal injections of carbolized water.

On the beginning of the fourth day the patient was suddenly seized with a severe chill, followed by the usual symptoms of septic poisoning, high temperature (104½°F.), rapid and feeble pulse, superficial respiration, great tympanites, thirst, vomiting, and arrested lochia, with no pain or tenderness over the abdomen. The outlook was anything but promising, but the prompt administration of large doses of quinine, combined with diaphoretics, turpentine stupes, warm fomentations, and the continued use of antiseptic injections was followed by the most gratifying results, and after four days of great anxiety the patient had recovered sufficiently to be declared out of danger. At the present time, eighteen days since the expulsion of the mole, the patient is up and about the house, with a good appetite, and making preparations to leave in a week or two on a journey to the south.

DISCUSSION.

PROFESSOR CHARLES WARRINGTON EARLE: I have seen two cases of this kind, and while I have been surprised a great many times in my practice, I was never more so than upon one of these occasions. I had been in practice about two years, when I was called to attend a lady in confinement near my residence. I placed myself at her bedside, found the *os uteri* well dilated, with the membranes intact and well down in the vagina, when all at once there came a gush of something, and a large quantity of these grape-like bodies made their appearance. I immediately gave ergot and cleared out the uterine cavity, and took the first opportunity to repair to my study to seek an explanation of this, at that time, to me a strange phenomenon. The case was eventually made the subject of a little article which appeared about that time in the *Chicago Medical Examiner.*

The lady was anæmic, and made a slow but perfect recovery. She has enjoyed good health since, but has never again become pregnant.

DR. HENRY T. BYFORD: I had an opportunity to see this specimen, and it was very much like a bunch of grapes in shape, although BARNES, I believe, claims there is no such resemblance. But he bases his views upon the fact that the vesicles are developed from each other instead of from the common stem.

In regard to the treatment, I think it would now be considered best to scoop out the uterus to prevent septicæmia, and so it would be, if that could be easily done. From inquiry of Doctor DOERING, I understand the opening in the cervix was rather small, the body anteverted, and it would have been necessary to use an instrument in removing the mole. I have seen severe inflammation in the broad ligaments result from curetting the uterus after abortions, with the dull curette. Therefore, I think it is a point of interest well illustrated in this case, that it is not in every instance the proper thing to do; especially when so much has been passed that there is pretty firm tonic contraction of the uterus.

DR. H. P. NEWMAN: I understood DR. DOERING to say he used ergot. This might explain the fact of finding the cervix closed.

DR. DOERING: I would like to ask DR. EARLE whether he discovered any trace of the fœtus.

PROFESSOR EARLE: I did not in my case, but there is a specimen in the museum of the College of Physicians and Surgeons in which the fœtus is one and one-half inches long.

DR. CHARLES CALDWELL: I met with one case in my practice last fall. October 10th, I was called early in the morning, the messenger informing me his wife was having a miscarriage. I found my patient flowing quite profusely. She supposed herself five months pregnant, as she had not menstruated since May.

The last week in July, she flowed slightly for two days. The 12th of August, the flow commenced again, and was so profuse that she went to bed and called a physician, who diagnosed her case threatened abortion, and treated her accordingly, keeping her in bed two weeks. From that time until the hydatidiform mole was expelled the flow never stopped completely, for a single day, but she passed no pieces of the mole. The os was soft and easily dilated. I introduced two fingers into the uterine cavity and removed its entire contents. The mass was too large to be removed intact, and the os was not sufficiently dilated. As I removed each piece the uterus contracted well and firmly, diminishing the size of its cavity rapidly, so I was sure when it was empty.

The lochial discharge kept up for three days. She remained in bed one day, but the next morning prepared her husband's breakfast, and has attended to her household duties since. There were no symptoms of septicæmia following. The broken mass would have filled a two quart measure. Some of the cysts were as large as a bean. I gave

PROFESSOR JAGGARD a specimen to show his class. No fœtus could be found in the mass. Small doses of ergot were given for a few days. Menstruation was established in December, and the patient is now strong and healthy.

III.—The inaugural thesis of PROFESSOR F. E. WAXHAM, M. D. (Chicago Medical College, 1878), entitled:

"INTUBATION OF THE LARYNX, WITH HISTORY OF CASES," was read by the SECRETARY, EDWARD WARREN SAWYER, M. D.

PROFESSOR WAXHAM described DR. O'DWYER's method of intubation of the larynx, narrated the histories of seventeen cases, in which the method had been employed, and drew the following conclusions:

"Intubation of the larynx possesses many advantages over tracheotomy:

"1. No opposition is met with on the part of parents and friends; quite a contrast to the difficulty which we usually meet in obtaining the consent to tracheotomy.

"2. It relieves the urgent dyspnœa as promptly and as effectually as tracheotomy, and if the child dies there is no regret that the operation was performed, and no discredit is attached to the physician.

"3. There is less irritation from the laryngeal tube than from the tracheal canula. As the tube is considerably smaller than the trachea, it does not press upon it firmly at any portion excepting at the chink of the glottis.

"4. Expectoration occurs more readily than through the tracheal tube.

"5. As the tube terminates in the throat, the air that enters the lungs is warm and moist from its course through the upper air passages, and there is less danger of pneumonia.

"6. It is a bloodless operation.

"7. It is more quickly performed and with less danger.

"8. There is no open wound that may be the source of constitutional infection.

"9. Convalescence is more rapid, as there is no ghastly wound to heal by slow granulations.

"10. The patient does not require the unremitting care of the physician, as in tracheotomy.

"11. I believe it to be a more successful method of treating croup, either diphtheritic or membranous, than tracheotomy.

"The only objection to the operation of intubation is the difficulty of its performance."

DISCUSSION.

PROFESSOR CHRISTIAN FENGER: MR. PRESIDENT, this is a subject of exceeding interest and deserves great attention. Tracheotomy is one of the few operations that always make me nervous. That this operation is attended with some danger there is no question. There is danger from hæmorrhage during and after the operation, and there is some danger of shock, which in cases where there is no membranous laryngitis, sometimes can be traced only to the operation. For instance, I years ago went to make an examination of an old man who had swallowed a little fish bone, that had got into the mucous membrane in the entrance to the larynx, during dinner. Œdema of the glottis necessitated tracheotomy, which was performed without an anæsthetic. There was no hæmorrhage, the dyspnœa was relieved, but the man died about twenty-four hours afterwards from the shock or disturbance attributable only and alone to the operation. In other cases hæmorrhage, even under great care in the operation, cannot always be avoided. I

have had two cases of such hæmorrhage where the patients have died, one in two hours and another in five hours subsequent to the operation, not on account of the amount of hæmorrhage, but on account of the disturbance in the lungs, caused by a moderate amount of aspirated blood. So if it is possible to get around the tracheotomy in some other way, then I for one would embrace it with the greatest of pleasure. However, when I saw PROFESSOR WAXHAM'S intubation—which I looked forward to with great interest—I got afraid of the tubes slipping into the air passages so I could not get hold of them again. That is one thing, and another that came into my mind was the small caliber of the opening in the tubes. I had an opportunity to see a little child that he operated upon where the operation was easy, and relief was instant, and that is all that I have seen as yet of the matter. Then, of course, I read the paper. There is one thing that I would be rather afraid of,—but that is a theoretical objection only,—that is, to leave the child without taking the tube out. It seems from the cases reported, however, that in those cases there has never been much trouble of this kind, and theory of any kind is of no value whatever compared with facts.

I believe that this matter is very worthy of careful consideration; but, on the other hand, not until a larger number of cases have been tried will we be able to form any definite opinion about it. But we know enough from the cases recorded here to be desirous of having this new matter tried.

PROFESSOR F. E. WAXHAM, M. D., was then elected Fellow of the Society.

W. W. JAGGARD, M. D., *Editor.*

22d February, 1886.

2330 Indiana Avenue.

